# Bystander Reactions to Workplace Incivility: The Role of Gender and Discrimination Claims

**DOI:** 10.5964/ejop.1675

**Published:** 2021-02-26

**Authors:** Samantha Sinclair

**Affiliations:** a Department of Psychology, Linnæus University, Växjö, Sweden; The Maria Grzegorzewska University, Warsaw, Poland

**Keywords:** discrimination attributions, gender bias, gender differences, emotional reactions, helping intentions, workplace incivility, discrimination prototypes

## Abstract

Will men and women receive the same support at work when they claim to have been discriminated against? This paper reports a scenario-based experimental study (N = 240, 50.4% women, M age = 25.65) that investigated bystanders’ reactions to an incident where a co-worker is treated in a condescending manner by another co-worker. The results showed that women reacted more strongly to the incivility incident and were more willing to support and defend the co-worker. As expected, the gender difference in helping intentions was especially prominent when the co-worker attributed the incident to gender discrimination, compared to a control condition with an attribution unrelated to gender. Further, when the incident was attributed to discrimination, the female co-worker evoked somewhat stronger helping intentions than the male co-worker, suggesting the presence of gender bias. The results are discussed in relation to the prototype perspective of perceived discrimination.

Previous research suggests that when people attribute negative events to discrimination, they tend to be negatively perceived. For example, a study by Kaiser and Miller (2001) found that an African American who failed a test was seen as a complainer when he attributed the failure to discrimination but not when he made other external attributions. Similarly, another study (Kaiser & Miller, 2003) found that an African American who attributed rejection on the labor market to discrimination was perceived as more irritating, even when discrimination was blatant. People have also been found to react more negatively to a person who blames a failing grade on discrimination, even when this person belongs to their ingroup (Garcia et al., 2005), and individuals who claim to have been discriminated can be perceived as more prejudiced against the outgroup (Blodorn & O’Brien, 2013). If individuals risk being negatively evaluated by others when they attribute events to discrimination, they may become more reluctant to make discrimination claims, which means that discrimination could continue unchallenged. Although there can be costs associated with making discrimination attributions, there may sometimes be benefits, such as receiving support from others. Unlike previous research on discrimination claims, the focus of the present paper is on prosocial reactions rather than social penalties. More specifically, the question is whether co-workers will evoke stronger reactions and helping intentions when they attribute being belittled by another co-worker to gender discrimination. The incident is an example of workplace incivility, which is defined as “low intensity, disrespectful or rude deviant workplace behavior with ambiguous intent to harm the target and is in violation of workplace norms for mutual respect” (Andersson & Pearson, 1999), and can be a precursor to workplace bullying (Baron & Nauman, 1996).

Although previous research have investigated bystanders’ reactions to alleged gender discrimination, this has primarily been done in the context of blatant ambient sexual harassment toward women (e.g., Bradley-Geist, Rivera, & Geringer, 2015; Chaudoir & Quinn, 2010; Hitlan, Schneider, & Walsh, 2006). While this work is indeed important, discrimination in the workplace based on gender and race is likely to have taken on more subtle forms in recent years and thus become more difficult to detect (Nier & Gaertner, 2012). For example, some individuals will claim to endorse egalitarian values while still expressing gender bias in the form of “selective incivility” (Kabat-Farr & Cortina, 2012). The present study therefore examined reactions to gender discrimination claims in a situation where there is no clear evidence pointing to discrimination. Furthermore, most studies on ambient sexism have not used a crossed design of participant and target gender, and without such a design, the constellations of possible gender bias that can be examined are limited. The present study used such a design to examine the nature of bystanders’ reactions to a co-worker who claim to have been discriminated against, and whether these reactions depend on bystander and co-worker gender.

## Discrimination Prototypes and Gender Bias

A recent study (Carlsson & Sinclair, 2018) showed that people tend to interpret a situation where a woman is rejected on the labor market as a sign of gender discrimination. This can be explained by a prototype account of perceived discrimination. In general, people will perceive events as discrimination to the extent that features of the event, such as the domain where it occurs, as well as the group membership of the victim and perpetrator, fit their preconception (“prototype”) of what a typical discrimination case looks like (Inman & Baron, 1996). Because women are prototypical victims of gender discrimination whereas men are not, it is possible that men who claim to be victims of gender discrimination end up receiving less support, which is a hypothesis that is put to the test in the current study. In other words, co-workers may receive different amounts of support depending on whether they are male or female. If such gender bias stems from discrimination prototypes, it should interact with the context. Specifically, this bias should be more evident in contexts where gender discrimination prototypes apply, such as sexual harassment, domestic violence, sexism, and the like. For example, female victims are met with more sympathy relative to male victims in the context of partner aggression (Hammock et al., 2017; Worthen & Varnado-Sullivan, 2005). In the present study, we thus expected that people would display more gender bias in the context of alleged gender discrimination, relative to a gender-neutral context.

Gender stereotypes portray women as more communal (helpful, understanding, and friendly, e.g., Bosak, Sczesny, & Eagly, 2008), and gender differences in self-reported communal traits have been observed across several cultures (Sinclair, Carlsson, & Björklund, 2016; Twenge, 1997, 2001). This gender difference seems pertinent in a workplace context, as women are more likely to endorse a relational approach to work, characterized by a belief that work is best accomplished through social relationships (Matthew, Buontempo, & Block, 2013). For example, women claim to engage in more interdependence oriented behaviors at work (Fletcher, 1999), and tend to deem potentially uncivil or harassing workplace behaviors as more offensive or inappropriate than men do (Montgomery, Kane, & Vance, 2004). Therefore, we expected women (relative to men) to be more prone to offer support to a co-worker who is subjected to workplace incivility.

Besides this general gender difference, women (relative to men) may display stronger reactions when a co-worker attributes an incivility incident to gender discrimination in particular. This hypothesis is based on the notion that women as a group have a history of being victims of gender discrimination and therefore may be more motivated to react to alleged gender discrimination in general. According to the defensive attribution theory (Shaver, 1970), whether an observer will sympathize with a victim is contingent on how personally relevant and potentially threatening the context is perceived. When observers feel that they are likely to be involved in a situation similar to that of the victim in the future, subsequent feelings of threat increases the potential for stronger reactions. Supporting this theory, Elkins, Phillips, and Konopaske (2002) found that women, but not men, felt threatened when faced with discrimination claims in an employment context, whereas men were instead more threatened in a child custody lawsuit context. Because female bystanders may anticipate a greater likelihood of becoming victims of future gender discrimination in organizations, we expected women (relative to men) to react more strongly in response to a co-worker who attributes being subjected to workplace incivility to gender discrimination, compared to an attribution that is gender neutral.

One can also imagine that women would be more motivated to help a co-worker who makes discrimination claims when this person is female rather than male, in other words, that they would exhibit a same-gender bias. However, based on the recent findings of Carlsson and Sinclair (2018), who with a total sample of almost 800, including a replication of Elkins and colleagues (Elkins, Phillips, Konopaske, & Townsend, 2001; Elkins et al., 2002), found virtually no evidence for same-gender bias in perceived gender discrimination, there is little reason to expect same-gender bias in the current study.

## The Present Study

This study is concerned with whether co-workers who attribute workplace incivility to gender discrimination end up evoking stronger reactions and helping intentions from bystanders, and further, whether this depends on co-worker and bystander gender. This research contributes to the literature by examining prosocial reactions to a person who makes discrimination claims, acknowledging that there could be pros and not only cons associated with making discrimination claims. Importantly, the crossed design of attribution type and participant and co-worker gender allows for putting gender bias in the form of victim prototype effects in reactions and helping intentions to the test. That is, whether men and women receive different levels of support in the case of gender discrimination attributions per se, or if there is a gender bias regardless of the co-worker’s attributions, which could be the case if women are regarded as victims in general (regardless of context). Moreover, previous studies do not reveal whether gender differences in reactions are unique to cases revolving around sexism per se (as defensive attribution theory would predict), or if women sympathize more with targets of unfair treatment in general, regardless of the nature of the conflict. The design was a 2 (co-worker’s attribution: gender discrimination vs. gender-neutral) × 2 (co-worker gender) × 2 (participant gender) between subjects factorial. The incivility incident was expected to elicit stronger emotions and helping intentions when the co-worker attributes the reason behind a rude colleague’s behavior to gender discrimination, compared to a gender-neutral attribution (Hypothesis 1). Second, women were expected to display stronger emotional reactions and exhibit stronger helping intentions overall, compared to men (Hypothesis 2). This gender difference was further predicted to interact with the co-worker’s attribution, such that women should (relative to men) differentiate more between the two contexts and react more when the co-worker attributes the incident to gender discrimination (Hypothesis 3). Finally, based on prototype theory, there should be an interaction between the co-worker’s attribution and co-worker gender, specifically, the incident should elicit stronger reactions when the co-worker is female and claims to have been discriminated against based on her gender (Hypothesis 4).

## Method

### Participants

Two-hundred and forty men and women (50.4% women, *M*_age_ = 25.65, *SD* = 7.54), recruited at public libraries and campuses in the south of Sweden, participated in the study without compensation. Work experience was a prerequisite to participate. The majority (78.8%) were students with work experience, 18.3% were employed full time and the remaining 2.9% did not report their occupation.

For reasons of transparency, we report how we determined the sample size, all data exclusions (if any), all manipulations, and all measures in the study. The sample size was selected to have about 80% power to detect moderate effects (*d* = 0.5) for the follow-up simple effects of H3 and H4, and we thus decided on *n* = 60 per cell (using G*power; Faul, Erdfelder, Lang, & Buchner, 2007). This also meant > 95% power to detect moderate effects in the analysis of variance (ANOVAs; $\eta_{p}^{2}$ = .13). Note that the design was not planned to have adequate power to follow-up a possible three-way interaction (as *n* = 30 per cell and *d* = 0.5 results in a power of 48%). Based on recent research on perceived discrimination (Carlsson & Sinclair, 2018) that found minimal same-gender bias effects, a very large sample would be required to follow up a three-way interaction. However, there was sufficient power to detect the possibility of a general same-gender bias in the form of a two-way interaction that would be manifested if bystanders were more upset and motivated to help in the case where the co-worker belongs to the same gender as themselves.

### Materials and Procedure

The study was introduced as investigating judgments of interpersonal relations in the workplace.

#### Experimental Manipulation

After being randomly assigned to conditions, the participants were handed a booklet that presented a scenario about a male or female upper secondary teacher who was belittled by his or her colleague:

Maria (Eric) works as an upper secondary teacher. One day a difficult situation presents itself at work. When Maria (Eric) suggests a new approach to solving the problem to a colleague, the colleague replies with a sneer: “What could you, who has only one year of experience, contribute with, while I on the other hand have been here for five years”.

The participants in the discrimination attribution condition then read the following sentence:

Maria (Eric) is convinced that the colleague behaves this way because the colleague has a problem with female (male) co-workers.

Participants in the control condition were instead presented with an attribution that was unrelated to gender:

Maria (Eric) is convinced that the colleague behaves this way because the colleague has a problem with new employees.

The participants were asked to imagine that they worked there and witnessed the event.

#### Emotional Reactions

The participants answered four questions that measured emotional reactions, specifically how upset, angry, sad, and dejected they felt about the incident (1 = *not at all*, 7 = *very much*, Cronbach’s α = .71).

#### Helping Intentions

Next, they responded to four statements that measured willingness to help and support the co-worker: “I would try to show Maria (Eric) my support by being extra nice to her,” “I would talk to Maria (Eric) and ask her how she is feeling after the incident,” “I would confront the colleague about their behavior,” and “I would report the incident to the person responsible for human resources” (1 = *no, definitely not*, 7 = *yes, definitely*). This measure had poor reliability (Cronbach’s α = .53). Omitting the item about being extra nice to Maria or Eric improved α to .62, however, the whole scale was used because omitting this item produced almost identical results. There was a moderate correlation between the emotional reactions scale and helping intentions scale, *r* = .40, *p* < .001.

#### Additional Measures

Apart from characteristics of the target, the extent to which discrimination prototypes are applied could also depend on characteristics of the perpetrator (Inman & Baron, 1996). After completing the outcome measures, the participants therefore provided brief descriptions of how they had imagined the rude colleague’s appearance, age, gender, and so on. We asked the question in this unobtrusive way because we were interested in whether people spontaneously imagine a male perpetrator, depending on co-worker (target) gender and discrimination claims. Finally, the participants answered demographic questions.

## Results

To test the four hypotheses, we conducted two three-way between groups ANOVAs (one for each dependent variable) with 2 (attributions: gender discrimination vs. gender-neutral) × 2 (co-worker gender) × 2 (participant gender). Means and standard deviations are presented in Table 1.

**Table 1:** Descriptive Statistics for Emotional Reactions and Helping Intentions

Experimental condition	Emotional reactions				Helping intentions			
	Female participants		Male participants		Female participants		Male participants	
	*M*	*SD*	*M*	*SD*	*M*	*SD*	*M*	*SD*
Discrimination attribution								
Female co-worker	4.97	1.07	4.08	1.16	5.53	0.85	4.55	1.05
Male co-worker	4.63	0.76	4.03	1.03	4.98	0.96	4.05	1.13
Gender-neutral attribution								
Female co-worker	4.33	1.06	3.82	1.09	4.66	0.99	4.18	0.92
Male co-worker	4.51	0.81	3.85	1.06	4.69	0.89	4.39	1.11

*Note*. *M* = mean; *SD* = standard deviation.

### Emotional Reactions

The ANOVA with emotional reactions as the dependent variable revealed a statistically significant main effect of attribution type with a weak effect size, *F*(1, 232) = 5.24, *p* = .02, $\eta_{p}^{2}$ = .022. This suggests that the participants reacted with more intense emotions when the co-worker attributed the incident to gender discrimination compared to an attribution that was unrelated to gender. This effect was significant for female participants, *t*(119) = −2.22, *p* = .028, *d* = −0.4, 95% CI [−0.76, −0.04], but not for male participants, *t*(117) = −1.11, *p* = .27, *d* = −0.05, 95% CI [−0.41, 0.31], providing partial support for Hypothesis 1.

Supporting Hypothesis 2, there was a medium sized main effect of participant gender, showing that women reacted with more negative emotions than men, *F*(1, 232) = 25.93, *p* < .001, $\eta_{p}^{2}$ = .10. This gender difference was significant in both the discrimination attribution condition, *t*(118) = 4.03, *p* < .001, *d* = 0.74, 95% CI [0.36, 1.11], and the gender-neutral condition, *t*(118) = 3.21, *p* = .002, *d* = 0.58, 95% CI [0.19, 0.97].

There was no significant main effect of co-worker gender, *F*(1, 232) = 0.09, *p* = .76, $\eta_{p}^{2}$ < .001, suggesting that the participants in general reacted with similar emotional intensity regardless of whether the co-worker was male or female.

The interaction effect between participant gender and attribution type, *F*(1, 232) = 0.38, *p* = .54, $\eta_{p}^{2}$ = .002, and between co-worker gender and attribution type, *F*(1, 232) = 1.26, *p* = .26, $\eta_{p}^{2}$ = .005, were non-significant, rendering no support for Hypotheses 3 and 4.

Finally, there was no significant interaction effect between participant gender and co-worker gender, *F*(1, 232) = 0.09, *p* = .77, $\eta_{p}^{2}$ < .005, indicating no same-gender bias, and the three-way interaction was also non-significant, *F*(1, 232) = 0.68, *p* = .41, $\eta_{p}^{2}$ = .003.

### Helping Intentions

The ANOVA with helping intentions as the dependent variable revealed a significant main effect of attribution type, which in line with Hypothesis 1 showed that a co-worker who made a discrimination attribution evoked stronger helping intentions, *F*(1, 232) = 5.35, *p* = .02, $\eta_{p}^{2}$ = .023. Similar to emotional reactions, this effect size was only significant for female participants, *t*(119) = −3.40, *p* = .001, *d* = −0.62, 95% CI [−0.99, −0.25], and not for male participants, *t*(117) = −0.08, *p* = .93, *d* = −0.02, 95% CI [−0.38, 0.34], providing partial support for the hypothesis.

Supporting Hypothesis 2, there was a significant and medium sized main effect of participant gender, as women expressed stronger intentions to help the co-worker, *F*(1, 232) = 27.23, *p* < .001, $\eta_{p}^{2}$ = .11. We confirmed that this gender difference was significant in both the discrimination attribution condition, *t*(118) = 5.05, *p* < .001, *d* = 0.92, 95% CI [0.54, 1.3], and the gender-neutral condition, *t*(118) = 2.19, *p* = .03, *d* = 0.4, 95% CI [0.04, 0.77].

**Figure 1:**
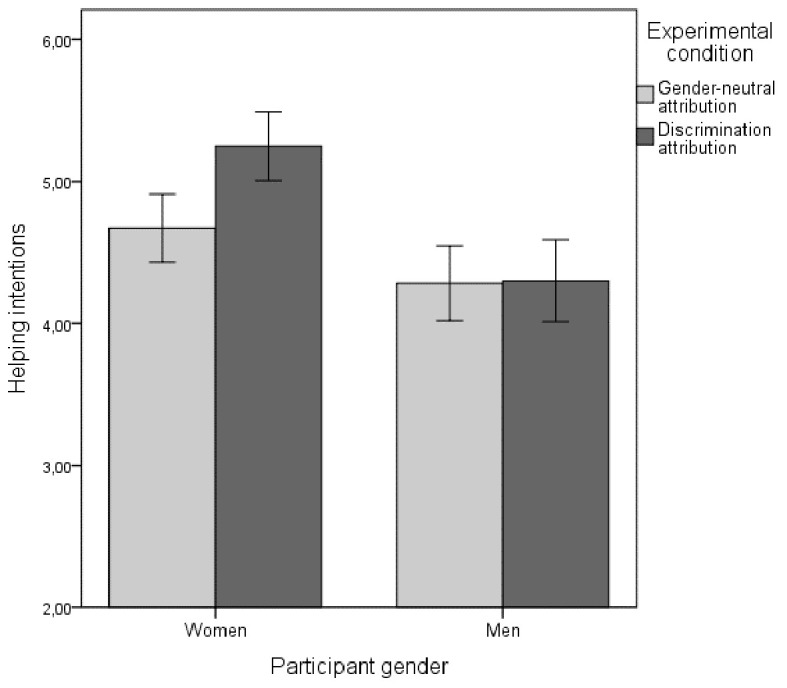
Effects of Attribution Type and Participant Gender on Helping Intentions *Note*. Error bars represent 95% confidence intervals.

There was also a statistically significant interaction effect between participant gender and attribution type, *F*(1, 232) = 4.85, *p* = .03, $\eta_{p}^{2}$ = .02, see Figure 1. That is, the gender difference in helping intentions was especially pronounced when the co-worker made a gender discrimination attribution, which is in line with Hypothesis 3. Follow-up *t*-tests showed that the gender difference was significant in both the discrimination attribution condition, *t*(118) = 5.05, *p* < .001, *d* = 0.92, 95% CI [0.54, 1.3], and the gender-neutral condition, *t*(118) = 2.19, *p* = .03, *d* = 0.4, 95% CI [0.04, 0.77], although the mean difference was more than twice as large in the discrimination attribution condition.

**Figure 2:**
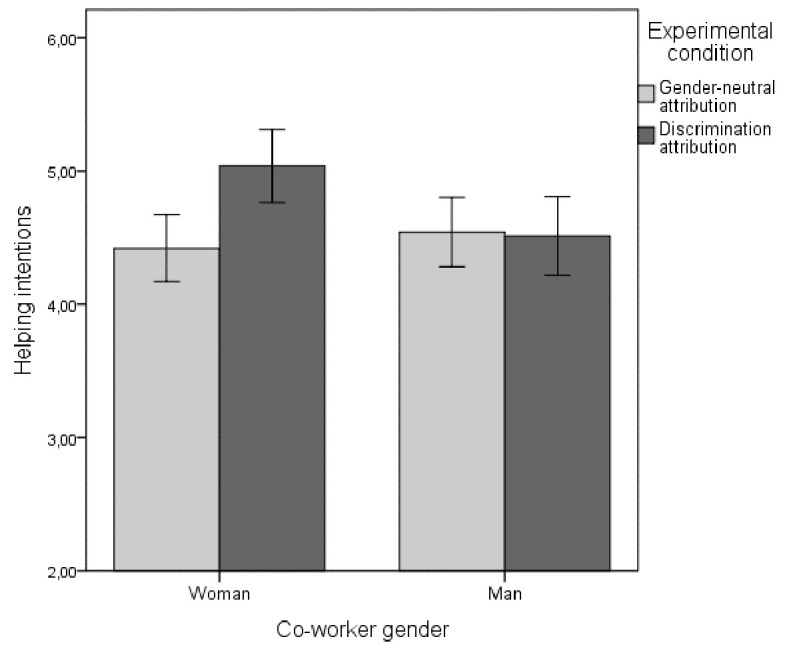
Effects of Attribution Type and Co-Worker Gender on Helping Intentions *Note*. Error bars represent 95% confidence intervals.

In line with Hypothesis 4, there was also a statistically significant interaction effect between attribution type and co-worker gender, as the participants were especially motivated to help the co-worker in the discrimination attribution condition when the co-worker was female, *F*(1, 232) = 6.26, *p* = .01, $\eta_{p}^{2}$ = .026. This interaction effect is displayed in Figure 2. Follow-up *t*-tests revealed that, as expected, the female co-worker received more help than the male co-worker in the discrimination attribution condition, *t*(118) = 2.60, *p* = .01, *d* = 0.48, 95% CI [0.11, 0.85], but there was no significant difference in the gender-neutral condition, *t*(118) = −0.67, *p* = .51, *d* = −0.12, 95% CI [−0.48, 0.24], suggesting the possibility that discrimination prototypes guide helping intentions.

As was the case with emotional reactions, the main effect of co-worker gender on helping intentions was non-significant, *F*(1, 232) = 2.55, *p* = .11, $\eta_{p}^{2}$ = .01, and so was the interaction between co-worker and participant gender, *F*(1, 232) = 0.20, *p* = .66, $\eta_{p}^{2}$ = .001, and the three-way interaction, *F*(1, 232) = 0.06, *p* = .80, $\eta_{p}^{2}$ < .001. This means that there were no indications of same-gender bias.

### Exploratory Analyses of How the Participants Imagined the Rude Colleague

We also conducted exploratory analyses on how the participants imagined the rude colleague in the scenario. Most participants (95.4%) provided descriptions of the colleague, and the majority (82.1%) mentioned gender. The answers were coded as mention versus no mention of a male colleague. An alternative coding (including only those who mentioned that the colleague was either male or female, and treating the rest as missing values) produced similar results.

**Figure 3:**
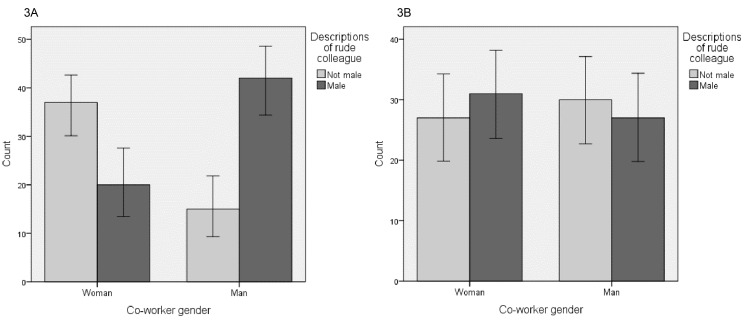
3A - Effect of Co-Worker Gender on Describing a Male Perpetrator in the Gender-Neutral Condition, 3B - Effect of Co-Worker Gender on Describing a Male Perpetrator in the Discrimination Attribution Condition *Note*. Error bars represent 95% confidence intervals.

A chi-square test for independence (with Yates Continuity Correction) revealed that in the gender-neutral attribution condition, the participants imagined a male colleague more often when the co-worker was male, χ²(1, *n* = 114) = 15.59, *p* < .01, ϕ = .39. This medium sized difference is displayed in Figure 3A. In the discrimination attribution condition, however, there was no apparent difference in mentioning a male colleague depending on gender of the co-worker, χ²(1, *n* = 115) = 0.22, *p* = .64, ϕ = −.06, see Figure 3B.

## Discussion

The present study examined whether co-workers who are subjected to workplace incivility evoke more or less reactions and helping intentions depending on whether they attribute the incident to gender discrimination, and further, how this interacts with co-worker and bystander gender. Previous research has primarily focused on the downsides to making discrimination attributions. The current findings add to this literature by showing that targets of workplace incivility may sometimes evoke stronger helping intentions when they make discrimination claims, at least when bystanders are female. Tentatively, this should encourage individuals who are victims of gender discrimination at the workplace to consider disclosing their experiences to a co-worker.

The results further revealed that when attributing the incivility incident to gender discrimination, the female co-worker evoked somewhat stronger helping intentions than the male co-worker. This is in line with the prototype perspective of perceived discrimination (Inman & Baron, 1996), which asserts that people perceive an act as discriminatory to the extent that it fits their image of a typical discrimination case (e.g., female victim). The findings suggest that discrimination prototypes may not only be important for perceptions but also for the way people treat each other in the workplace. However, this effect was not significant for emotional reactions, spurring the need for further research. The very weak (and insignificant) main effects of co-worker gender suggest that women are not necessarily treated as victims in incivility contexts in general, but rather may be more readily supported as victims in gender discrimination contexts per se. Moreover, that men and women did not seem to be selectively supporting of co-workers of their own gender suggests that there was little or no same-gender bias, which is similar to recent findings on perceived gender discrimination (Carlsson & Sinclair, 2018).

Akin to previous findings (Montgomery et al., 2004), we also found that women displayed stronger reactions to a case of workplace incivility, compared to men. This can perhaps be explained by women having a more relational approach to work (Matthew et al., 2013). Women were especially motivated to help the co-worker when the co-worker made discrimination attributions compared to a gender-neutral attribution, regardless of co-worker gender. This suggests that women may be particularly motivated to detect and react against discrimination, which is in line with defensive attribution theory (Shaver, 1970). However, this interaction effect was statistically significant for helping intentions but not for emotional reactions and as such the hypothesis was only partially supported.

The explorative analyses suggested that in the gender-neutral (control) condition, it was common to think of the rude colleague (“perpetrator”) as belonging to the same gender as the co-worker (“target”). In the discrimination attribution condition however, the proportions seem to shift from same-gender dyads into more cases of other-gender dyads, which might be due to activation of discrimination prototypes. Although the upper secondary teacher occupation is gender balanced in Sweden, there is a possibility that this occupation is associated more with females, and that this influenced the imagined perpetrator. It is also possible that if the perpetrator were a manager instead of a peer, other gender constellations would more readily come to mind.

### Practical Implications

Because it can be difficult to determine the severity of a workplace conflict, and whether it involves illegal behaviors such as discrimination, managers need to take multiple perspectives into consideration, including those of the victim, the alleged harasser, and other individuals in the work environment such as bystanders (Raver & Gelfand, 2005). The finding that female bystanders may become more motivated to offer support when a conflict is attributed to gender discrimination rather than being new at the job could be regarded as reasonable: If a colleague has problems with people because they are new on the job, this conflict may be more likely to dissipate over time. Because a person’s gender in most cases remains constant, there could be more reason for bystanders to perceive the conflict as serious in this case. However, the observed gender difference in reactions and helping intentions may have implications for women’s stress levels: If they have a lower threshold to perceive incivility as serious, female bystanders may experience higher levels of stress reactions when there is an interpersonal conflict at the workplace. On a positive note, that bystanders care is good news for victims of workplace incivility: If employees fail to react when observing incivility and harassment, incivility could become the norm, which might foster an organizational culture of conflict (Pearson, Andersson, & Porath, 2005).

### Limitations and Directions for Future Research

In the gender-neutral condition, the co-worker attributed the colleague’s rude behavior to being new on the job. Because discrimination might be perceived as more serious when there is no group permeability, that is, when switching groups is not possible (Lalonde & Silverman, 1994), and because being new on the job is temporary, future research may want to examine reactions to co-workers’ attributions to gender discrimination compared to attributions to, for example, discrimination due to ethnicity or age.

It would also be optimal to experimentally vary gender of the perpetrator, which could not be accomplished in the current study, as it would have meant increasing the experimental design. Future studies may however prefer to hold this variable fixed rather than unknown, in order to gain more control over the way that participants imagine gender constellations in interpersonal conflicts.

Another limitation of this study was the low reliability in one of the outcome measures (helping intentions). Although the low reliability makes it less likely to find an effect, and thus should decrease the risk of making a Type I error through decreased power, future research on this topic would be wise to use a more reliable measure of helping intentions.

Our incivility scenario corresponds to two out of the three types of bullying behaviors that seem to be especially related to stress reactions among employees, specifically, judging someone’s work unjustly or in an offending manner, and limiting someone’s possibilities of expressing his or her opinions (Vartia, 2001). This suggests that the scenario bears resemblance to a realistic workplace incident in real life. For reasons of external validity, we included only participants with work experience, who could relate to the scenario. Nevertheless, considering that behavioral intentions sometimes do not correspond perfectly with real behavior (e.g., Kraus, 1995; Wojciszke & Bocian, 2018) it would be ideal to replicate the study in an authentic workplace situation using behavioral measures, preferably of real helping behavior.

The present research was conducted in Sweden and the findings should preferably be replicated in other cultures. For example, a recent meta-analysis by Triana, Jayasinghe, Pieper, Delgado, and Li (2018) found stronger associations between perceived gender discrimination and poorer health and work-related outcomes in countries with relatively high gender equality. It is thus possible that people react differently to a co-worker’s discrimination claims depending on culture.

On a final note on generalizability, because the current study investigated reactions to an incident in a gender-balanced occupation, it may not generalize to male- and/or female-dominated occupations. However, previous research (Carlsson & Sinclair, 2018) has found that prototype effects in perceived gender discrimination are strongest in male-typed occupations and weakest in female-typed occupations. We therefore suspect that prototype effects in reactions to discrimination claims may follow the same pattern.
